# B细胞非霍奇金淋巴瘤合并乙型肝炎病毒感染患者的临床特征及病毒再激活

**DOI:** 10.3760/cma.j.issn.0253-2727.2021.06.013

**Published:** 2021-06

**Authors:** 艳秋 徐, 伟 陈, 汉影 沈, 舒蕾 董, 君 李, 开然 李, 振宇 李, 锋 朱, 开林 徐

**Affiliations:** 1 徐州医科大学血液病研究所 221004 Blood Disease Institute, Xuzhou Maedical University, Xuzhou 221004, China; 2 徐州医科大学附属医院血液科 221006 Department of Hematology, The Affiliated Hospital of Xuzhou Medical University, Xuzhou 221006, China

淋巴瘤是起源于淋巴结和其他淋巴组织的恶性肿瘤，其中非霍奇金淋巴瘤（NHL）较为常见[1]。我国是乙型肝炎病毒（HBV）感染高发地区，约有1.7亿慢性HBV携带者[2]–[3]。相关流行病学研究认为B细胞NHL（B-NHL）与HBV感染密切相关，HBV感染者发生NHL的风险约为非感染者的2～3倍[3]。合并HBV感染的淋巴瘤患者化疗过程中易发生HBV再激活，若诊治不恰当，不仅影响疗效，甚至会造成患者死于HBV再激活引起的肝衰竭[4]。为此我们回顾性分析近年来我院收治的231例B-NHL患者的临床资料，探讨B-NHL合并HBV感染患者的临床特征、预后及HBV再激活的相关因素，以期为临床提供借鉴和帮助。

## 病例与方法

1. 病例：选取自2012年12月至2019年10月徐州医科大学附属医院血液科收治的231例B-NHL患者，中位年龄为58（17～93）岁。纳入标准：①病理学及免疫组化符合B-NHL的诊断[5]；②初次入院“乙肝两对半”检查结果为HBV现症感染［乙型肝炎表面抗原（HBsAg）阳性］或既往感染［HBsAg阴性且乙型肝炎核心抗体（HBcAb）阳性］；③至少接受3个疗程化疗。排除标准：患者合并甲型、丙型、丁型、戊型肝炎病毒及人类免疫缺陷病毒等感染或其他类型肿瘤。

2. 临床资料：采集的一般临床资料包括年龄、IPI评分、结外侵犯数目等。实验室检查包括初诊时的血清乳酸脱氢酶（LDH）、血清β_2_-微球蛋白（β_2_-MG）、红细胞沉降率（ESR）、HBV血清标志物［HBsAg、乙型肝炎表面抗体（HBsAb）、乙型肝炎e抗体（HBeAb）、乙型肝炎e抗原（HBeAg）和HBcAb］、肝功能、骨髓检查等。

3. HBV再激活的评判标准[6]–[8]：①HBsAg阳性且符合下列任一条件：血清HBV DNA由不可测变为可测或超过基线水平10倍；HBeAg阴性者血清转阳。②HBsAg阴性且符合下列任一条件：HBsAg转阳；血清HBV DNA由不可测变为可测。结果判读：HBV DNA≥1×10^3^拷贝/ml定义为阳性组，HBV DNA≥1×10^5^拷贝/ml定义为高拷贝组，HBV DNA≥1×10^3^拷贝/ml且<1×10^5^拷贝/ml定义为HBV DNA低拷贝组[9]；ALT水平增加3倍或者超过100 IU/L定义为HBV相关肝炎暴发[10]。

4. 疾病分期：采用Ann Arbor分期标准[11]，按有无B症状分为A组和B组，按照国际预后指数（IPI）评分标准对患者进行评分[12]。

5. 疗效评价及随访：疗效评价标准[13]：①完全缓解：所有病灶证据均消失；②部分缓解：可测量病灶缩小，无新病灶；③疾病稳定：未完全缓解、部分缓解，无疾病进展；④复发或疾病进展：任何新增病灶或原病灶直径增加≥50％。以电话、住院病历等方式进行随访，记录患者的生存情况。无进展生存（PFS）期：自开始化疗到淋巴瘤进展、因任何原因死亡或末次随访时间。总生存（OS）期：自确诊至因任何原因死亡或末次随访时间。随访日期截至2020年5月，中位随访时间为35个月。

6. 统计学处理：利用SPSS 22.0软件进行统计学分析，分类变量用例数（百分比）表示，计数资料组间比较采用*χ*^2^检验，使用Kaplan-Meier法绘制生存曲线，*P*<0.05为差异有统计学意义。

## 结果

1. 一般临床特征：入组的231例患者中，男131例，女100例，中位年龄58（17～93）岁。DLBCL患者144例（62.3％），滤泡性淋巴瘤（FL）患者25例（10.8％），黏膜相关淋巴组织（MALT）淋巴瘤患者24例（10.4％），套细胞淋巴瘤（MCL）患者9例（3.9％），其他B细胞淋巴瘤患者29例（12.6％）；Ann Arbor分期Ⅰ～Ⅱ、Ⅲ～Ⅳ期患者分别为88例（38.1％）、143例（61.9％）；IPI评分0～2分、3～5分患者分别有160例（69.3％）、71例（30.7％）；LDH水平升高者106例（45.9％）；β_2_-MG水平升高者102例（44.2％）；有B症状者54例（23.4％）；结外受累区域≥2个者33例（14.3％）；肝脏受累者11例（4.8％）。

61例患者为HBV现症感染，170例患者为HBV既往感染，与既往感染组比较，HBV现症感染组患者年龄<60岁、LDH及β_2_-MG水平升高、IPI评分3～5分、结外受累区域≥2个、肝脏受累、HBV再激活的比例更高，差异均有统计学意义（*P*值均<0.05）（表1）。

**表1:** 231例现症与既往感染乙型肝炎病毒（HBV）的B细胞非霍奇金淋巴瘤患者临床特征比较［例（％）］

临床特征	HBV现症感染（61例）	HBV既往感染（170例）	*χ*^2^值	*P*值
年龄			11.874	0.001
<60岁	44（72.1）	79（46.5）		
≥60岁	17（27.9）	91（53.5）		
LDH水平			22.336	<0.001
升高	44（72.1）	62（36.5）		
正常	17（27.9）	106（62.4）		
缺失数据	0（0）	2（1.1）		
β_2_-微球蛋白水平			17.060	<0.001
升高	40（65.6）	62（36.5）		
正常	21（34.4）	97（57.1）		
缺失数据	0（0）	11（6.4）		
IPI评分			50.394	<0.001
0～2分	43（70.5）	142（83.5）		
3～5分	18（29.5）	28（16.5）		
结外侵犯数目			15.686	<0.001
<2	43（70.5）	155（91.2）		
≥2	18（29.5）	15（8.8）		
肝脏受累			12.752	<0.001
是	8（13.1）	3（1.8）		
否	53（86.9）	167（98.2）		
骨髓受累			0.105	0.746
是	7（11.5）	17（10.0）		
否	54（88.5）	153（90.0）		
肝炎暴发			2.198	0.138
是	17（27.9）	32（18.8）		
否	44（72.1）	138（81.2）		
HBV再激活			71.837	<0.001
是	27（44.3）	7（5.9）		
否	34（55.7）	163（94.1）		
末次化疗疗效			10.944	0.012
完全缓解	13（21.3）	60（35.3）		
部分缓解	12（19.7）	42（24.7）		
疾病稳定	1（1.6）	10（5.9）		
疾病进展	35（57.4）	58（34.1）		

2. HBV再激活的相关因素：61例HBV现症感染者中27例（44.3％）发生HBV再激活，HBV再激活的关键因素为化疗前未行抗HBV治疗（48.1％对17.6％，*P*＝0.011）。170例HBV既往感染者中，7例（4.1％）发生HBV再激活，与HBV未激活组比较，HBV再激活组骨髓受累（42.9％对8.6％，*P*＝0.021）、应用利妥昔单抗（100％对62％，*P*＝0.049）、HBsAb滴度<100 IU/L（100％对41.1％，*P*＝0.003）的患者比例更高。

3. HBV再激活患者的临床特征：入组的231例患者中有34例发生HBV再激活，其中27例（79.4％）为HBV现症感染者，7例（20.6％）为HBV既往感染者，两组预防性抗病毒患者比例差异有统计学意义（51.9％对0，*P*＝0.014），既往感染组中发生HBV再激活的患者均未预防性抗病毒治疗。

4. HBV表达水平与临床特征的相关性：本研究的231例患者中，105例进行了HBV DNA检测。其中HBV DNA阳性者47例（44.8％），阴性者58例（55.2％）。HBV DNA阳性组与阴性组比较，LDH水平升高（74.5％对51.7％，*P*＝0.017）、β_2_-MG水平升高（74.5％对17.2％，*P*<0.001）、肝脏受累（14.9％对3.4％，*P*＝0.037）、HBsAb<100 IU/L（97.9％对62.1％，*P*<0.001）、肝炎暴发（36.2％对15.5％，*P*＝0.015）、HBV再激活（53.2％对1.7％，*P*<0.001）的患者比例差异具有统计学意义。以1×10^5^拷贝/ml为界值将HBV DNA阳性患者划分为HBV DNA高拷贝组（25例）和低拷贝组（22例），高拷贝组ESR水平升高者比例较低拷贝组高（72％对13.6％），差异具有统计学意义（*P*<0.001）。

5. 化疗的疗效：选择末次化疗进行疗效评估，与HBV现症感染组比较，HBV既往感染组完全缓解率、部分缓解率、疾病稳定率高，疾病进展率低（*P*＝0.012）（表1）。HBV现症感染者中，与HBV再激活组比较，HBV未激活组完全缓解率（23.5％对22.2％）、部分缓解率（32.4％对11.1％）高，疾病进展率（41.2％对59.3％）低（*P*＝0.050）；HBV既往感染者中，HBV再激活组与未激活组比较，化疗应答情况的差异无统计学意义（*P*＝0.197）。发生HBV再激活的患者中，HBV现症感染组与HBV既往感染组比较，化疗应答情况的差异无统计学意义（*P*＝0.835）。HBV DNA阳性组与阴性组比较，化疗应答情况的差异无统计学意义（*P*＝0.695），HBV DNA高拷贝组与低拷贝组化疗应答情况的差异无统计学意义（*P*＝0.418）。

6. 生存预后分析：本次回顾性研究中，Kaplan-Meier分析显示HBV现症感染组与既往感染组比较，PFS（*P*＝0.118）及OS（*P*＝0.333）的差异无统计学意义。HBV再激活组与未激活组比较，PFS（*P*＝0.231）及OS（*P*＝0.558）的差异无统计学意义。HBV DNA阳性组与阴性组比较，PFS（*P*＝0.445）及OS（*P*＝0.823）的差异亦无统计学意义。HBV DNA低拷贝组较高拷贝组具有更好的PFS（*P*＝0.040），但OS的差异无统计学意义（*P*＝0.556）。

## 讨论

本研究发现，与HBV既往感染者相比，现症感染者更年轻，LDH及β_2_-MG水平升高，IPI评分高，结外受累部位多，肝脏易受累，与既往研究结果一致[14]–[15]。HBV既往感染组的缓解率高，疾病进展率低（*P*＝0.012），表明合并HBV现症感染的B-NHL患者化疗效果差。有研究证实，合并HBV现症感染的B-NHL患者OS及PFS时间较HBsAg阴性者缩短[15]–[16]，而我们研究发现HBV现症感染组与既往感染组比较，PFS及OS的差异均无统计学意义，似乎与既往研究结果不一致，可能与我们设定的对照组是合并HBV既往感染（HBsAg阴性且HBcAb阳性）的淋巴瘤患者有关。只要感染过HBV，无论病毒是否被清除，HBcAb多为阳性，若存在的微量病毒未被完全清除，而实验室指标无法检测，可能会造成研究结果出现差异。

大量研究表明，HBV现症感染者是HBV再激活的高危人群，化疗时应预防性抗病毒治疗[17]–[18]。本研究也证实，与HBV既往感染组相比，现症感染组HBV再激活发生率高（*P*<0.001），HBV现症感染者中未激活组预防性抗病毒比例较高（*P*＝0.011），这也提示对于HBV现症感染患者，尽早抗病毒治疗是防止HBV再激活的关键措施。对于HBsAg阴性NHL患者，相关研究认为利妥昔单抗、类固醇激素联合化疗是HBV再激活的危险因素，但是否需要预防性抗病毒尚未建立统一标准[15]–[16],[19]。有研究认为HBcAb是病毒再激活的独立危险因素，对于HBV既往感染患者，预防性抗病毒治疗是有益的[18]。本研究入组的231例患者中有34例发生HBV再激活，其中HBV既往感染者7例（4.1％），化疗方案均是利妥昔单抗联合类固醇激素，且均未预防性抗病毒治疗。这也提示，对于HBsAg阴性患者，HBcAb阳性、应用利妥昔单抗是HBV再激活的危险因素，预防性抗病毒治疗对于HBV既往感染者是有益的。更多研究考虑到耐药性和成本问题，倾向于监测HBV DNA水平，当HBV DNA水平可测时应及早干预[6]–[9]。

HBV再激活组与未激活组比较，PFS（*P*＝0.231）及OS（*P*＝0.558）的差异无统计学意义。Su等[20]的回顾性研究表明，HBV再激活不会影响患者的PFS，但会缩短OS时间，HBV再激活可能会影响疗效，延长化疗周期，从而增加淋巴瘤进展风险，影响患者的OS。本研究纳入的病例数较少，具有一定局限性，需要进一步扩大样本量。

HBV DNA是病毒复制指标之一，是抗病毒治疗及疗效判断的重要指标[8]。本次回顾性研究中，HBV DNA阳性患者易出现肝脏受累、肝炎暴发，HBV再激活发生率高。与HBV DNA高拷贝组相比，低拷贝组具有更好的PFS（*P*＝0.040），这也证实HBV DNA复制特别是高复制状态会影响疗效，因此在HBV病毒复制情况下，患者需积极抗病毒治疗，不仅可减轻肝脏受损情况，也能防止HBV再激活发生。

综上，与HBV既往感染者相比，合并HBV现症感染的B-NHL患者疗效及预后情况较差，易发生HBV再激活。HBV DNA高拷贝组患者的预后情况较低拷贝组差。此外，对于HBV既往感染患者，联合利妥昔单抗化疗时发生HBV再激活的风险高，需严格监测患者乙型肝炎病毒标志物、HBV DNA、肝功能等指标，及时干预。本研究为回顾性研究，样本量偏小，随访时间较短，有待进一步行前瞻性研究并深入探讨相关机制。
